# ﻿*Hivanua*, a new genus of harmochirine jumping spiders from the Marquesas Islands (Araneae, Salticidae, Harmochirina)

**DOI:** 10.3897/zookeys.1200.120868

**Published:** 2024-05-09

**Authors:** Wayne P. Maddison

**Affiliations:** 1 Departments of Zoology and Botany and Beaty Biodiversity Museum, University of British Columbia, 6270 University Boulevard, Vancouver, British Columbia, V6T 1Z4, Canada University of British Columbia Vancouver Canada

**Keywords:** Classification, molecular phylogeny, new species, Plexippini, Salticoida

## Abstract

The genus *Hivanua***gen. nov.** is established for the harmochirine jumping spiders of the Marquesas Islands, formerly placed in *Habronattus* F.O. Pickard-Cambridge, 1901 and *Havaika* Prószyński, 2002. The type species, *Hivanuatekao***sp. nov.** is described, and five species described by Berland are re-illustrated and moved into the genus: *Hivanuaflavipes* (Berland, 1933), **comb. nov.**, *Hivanuanigrescens* (Berland, 1933), **comb. nov.**, *Hivanuanigrolineata* (Berland, 1933), **comb. nov.**, *Hivanuarufescens* (Berland, 1934), **comb. nov.**, and *Hivanuatriangulifera* (Berland, 1933), **comb. nov.** The female epigyne is much like that of *Habronattus*, *Bianor* Peckham & Peckham, 1896, and other harmochirines, with a centrally placed coupling pocket and two atria with crescent-shaped edges. The terminal apophysis of the male palp, which is variable throughout the pellenine subgroup of the Harmochirina, is absent in *H.rufescens* but present in *H.tekao***sp. nov.**, in which it is elbowed much as in *Habronattus*. These Pacific Island harmochirines, like the *Havaika* of Hawaii, appear to be largely foliage dwellers, unlike most of their continental relatives.

## ﻿Introduction

Among the jumping spiders on islands of the central Pacific are a few species of the pellenine clade of the subtribe Harmochirina (Maddison 2015), a group well known elsewhere for *Habronattus* F.O. Pickard-Cambridge, 1901 (in the Americas) and *Pellenes* Simon, 1876 (mostly in Afro-Eurasia). These central Pacific harmochirines include 23 described species of the genus *Havaika* Prószyński, 2002 in Hawaii (Simon 1900; Prószyński 2002, 2008; Arnedo and Gillespie 2006), and further south, in the Marquesas Islands of French Polynesia, a few species that have been placed in *Havaika* and *Habronattus* (Berland 1933, 1934; Prószyński 2002). The species of Hawaii and the Marquesas share some traits unusual among harmochirines: they appear to be mostly vegetation-dwelling (most harmochirines are ground-dwellers), they correspondingly have traits usually seen only in vegetation-dwellers of their size (scales with a sheen; legs with sparse setation), and their described species lack a terminal apophysis in the male palp (generally present in pellenine harmochirines, except for *Neaetha* Simon, 1884 and some PellenessubgenusPellenattus Maddison, 2017). Recent work on the molecular phylogeny of harmochirines by Azevedo et al. (2024, in press), confirmed here, shows that the Hawaiian and Marquesan lineages do not form a clade, but rather that the Marquesan lineage is the sister group to *Pellenattus*, while the Hawaiian *Havaika* is the sister group to *Habronattus*+*Pellenattus*+the Marquesan lineage. The Marquesan lineage therefore needs to be moved out of *Havaika*. Accordingly, it is here described as the new genus *Hivanua* Maddison gen. nov., containing six recognized species, one of which is new and has a *Habronattus*-like terminal apophysis.

## ﻿Material and methods

### ﻿Material examined

Spider specimens examined morphologically for this study are deposited in the Bernice P. Bishop Museum (**BPBM**), the Essig Museum of the University of California, Berkeley (**EMEC**), and the Natural History Museum, London (**NHMUK**).

### ﻿Morphology

Preserved specimens were examined under both dissecting microscopes and a compound microscope with reflected light. Drawings were made with a drawing tube on a Nikon ME600L compound microscope. Microscope photographs were made on an Olympus SZX12 stereoscope and focus-stacked using Helicon Focus v. 4.2.7.

All measurements are given in millimeters. Descriptions of color pattern are based on the alcohol-preserved specimen. Carapace length was measured from the base of the anterior median eyes not including the lenses to the rear margin of the carapace medially; abdomen length to the end of the anal tubercle. The following abbreviations are used: **ALE**, anterior lateral eyes; **ECP**, epigynal coupling pocket; **PLE**, posterior lateral eyes; **PME**, posterior median eyes (the “small eyes”); **RTA**, retrolateral tibial apophysis; **TmA**, terminal apophysis.

### ﻿Molecular data and phylogenetic analysis

To understand species distinctions in the genus *Hivanua*, and to test whether the two sampled Marquesan species (*H.tekao* sp. nov. and *H.rufescens*) form a clade, molecular data were newly gathered from 17 *Hivanua* and other harmochrine specimens by Ultraconserved Element (UCE) target enrichment sequencing methods (Faircloth 2017), using the RTA (Zhang et al. 2023) and Spider (Kulkarni et al. 2019) probesets, and with the assistance of Arbor Biosciences. These data were combined with data for five taxa obtained by similar methods by Azevedo et al. (2024, in press) to assemble a UCE dataset of 22 species (Table 1). *Bianor* serves as the outgroup because it is not from the pellenine subgroup, but from the *Harmochirus* subgroup of harmochirines (Maddison 2015). Molecular protocols followed those of Marathe et al. (2024).

**Table 1.** Specimens in molecular phylogeny. j. = juvenile or penultimate instar. Sequence Read Archive (SRA) accession numbers with * indicate data from Azevedo et al. (2024, in press). Nuku Hiva and Hiva Oa are in the Marquesas Islands of French Polynesia. Last three columns show number of UCE loci, and total sequence length in base pairs (bps) for UCE loci and mitochondrial genome.

Species	Specimen ID		Probeset	SRA#	Location	GPS Coordinates (Latitude, Longitude)	Reads pass QC	UCEs	UCE bps	mt bps
*Bianormaculatus* (Keyserling, 1883)	NZ19.9864	♂	RTA	SAMN40752353	New Zealand: Canterbury	-42.1691, 172.8090	7914001	1347	1407035	
*Habronattuscoecatus* (Hentz, 1846)	d210	♂	RTA	SAMN40752354	USA: Texas: Rio Grande City	26.5000, -98.8751	700294	1258	677212	—
*Habronattuscontingens* (Chn., 1925)	G3303	♀	Spider	SAMN39938211*	Mexico: Jalisco: Zapopan	20.6897, -103.6104	3050339	696	526233	7452
*Habronattushirsutus* (P. & P., 1888)	IDWM.21018	♂	RTA	SAMN40752355	Canada: British Columbia: Mayne I.	48.827, -123.265	3951254	1343	1380291	13037
*Habronattusophrys* Griswold, 1987	IDWM.21006	♀	RTA	SAMN40752356	Canada: British Columbia: Mayne I.	48.8221, -123.2627	3951254	1398	1488825	14467
*Havaikajamiesoni* Prószyński, 2002	IDWM.21009	♂	Spider	SAMN40752357	USA: Hawaii: Kauai: Kōke’e	22.1172, -159.6697	3951254	722	620643	14293
*Havaika* cf, *kauaiensis* Prószyński, 2002	IDWM.21010	♂	RTA	SAMN40752358	USA: Hawaii: Kauai: Kōke’e	22.1252, -159.6645	3951254	1356	1402805	14479
*Hivanuarufescens* (Berland, 1934)	IDWM.22080	j.	RTA	SAMN40752359	Hiva Oa, Temetiu Ridge	-9.81, -139.08	3951254	1013	360939	14434
*Hivanuarufescens* (Berland, 1934)	IDWM.23002	j.	RTA	SAMN40752360	Hiva Oa, Temetiu Ridge	-9.81, -139.08	3951254	1222	445199	3537
*Hivanuatekao* sp. nov.	d560	♂	Spider	SAMN39938226*	Nuku Hiva, Mt Tekao	-8.9, -140.2	495722	182	52408	2568
*Hivanuatekao* sp. nov.	IDWM.23001	♀	RTA	SAMN40752361	Nuku Hiva, Mt Tekao	-8.9, -140.2	3951254	1444	821685	4464
Hivanuacf.tekao	d558	♀	Spider	SAMN39938225*	Nuku Hiva, Mt Tekao	-8.9, -140.2	1077488	765	445228	—
Hivanuacf.tekao	d561	♂	Spider	SAMN39938224*	Nuku Hiva, Mt Tekao	-8.9, -140.2	618949	298	91230	12708
Hivanuacf.tekao	IDWM.22077	j.♂	Spider	SAMN40752362	Nuku Hiva, Mt Tekao	-8.9, -140.2	16024385	363	119803	14363
Hivanuacf.tekao	IDWM.22078	j.♂	Spider	SAMN40752363	Nuku Hiva, Mt Tekao	-8.9, -140.2	18273055	325	101913	14364
Hivanuacf.tekao	IDWM.22079	♀	Spider	SAMN40752364	Nuku Hiva, Mt Tekao	-8.9, -140.2	24724214	639	292126	14362
Pellenesaff.crandalli (L.&G. 1955)	IDWM.23003	♀	RTA	SAMN40752365	USA: Colorado: Berthoud	40.3, -105.1	5001755	1326	1449221	—
Pellenesaff.longimanus (Em. 1913)	G2972	♀	RTA	SAMN40752366	USA: Texas: N Rio Grande City	26.5000, -98.8751	6555856	1322	1424528	—
*Pellenespeninsularis* (Emerton, 1925)	d555	j.♂	Spider	SAMN40752367	Canada: Ontario: Dwight	45.3384, -79.0302	1609418	733	533368	—
*Pellenesshoshonensis* (Gertsch, 1934)	NA19.1434	♂	RTA	SAMN40752368	USA: Washington: Columbia NWR	46.937, -119.247	3304592	1358	1385614	—
*Pelleneswashonus* (L.&G. 1955)	IDWM.21013	♂	Spider	SAMN40752369	USA: California: Pepperwood Pres.	38.57, -122.69	10334966	742	640000	13734
*Pellenestripunctatus* (Wlck., 1802)	d556	♀	Spider	SAMN39938245*	Germany: Saxony, Authausen	51.607, 12.711	6546246	709	577816	—

UCE loci were identified among the assembled contigs using the RTA probset file and PHYLUCE (Faircloth 2016). After recovery, each locus was realigned with MAFFT v. 7.505 (Katoh and Standley 2013) using the LINSI option. Poorly aligned areas were deleted using GBLOCKS (Castresana 2000; Talavera and Castresana 2007) as implemented in Mesquite v. 3.81 (Maddison and Maddison 2023b), with parameters as follows: min. fraction identical at conserved = 0.5, min. at highly conserved = 0.7, counting fraction only within taxa with non-gaps at that position; max. length of non-conserved blocks = 8, min. length of block = 8, fraction of gaps allowed = 0.6. Loci were retained for analysis only if they were recovered in at least 3 *Hivanua* specimens and in at least 10 taxa total, and if a preliminary RAxML (Stamatakis 2014) gene tree had the ratio of the two longest branches less than 5, to guard against paralogy (see Maddison et al. 2020b). Mitochondrial genes were found among the contigs by BLAST as described by Maddison et al. (2020a), using the mitochondrial genome of *Habronattusoregonensis* (Peckham and Peckham 1888) (Masta and Boore 2004) as target. The mitochondrial genomes were aligned by MAFFT using the LINSI option.

Maximum-likelihood phylogenetic analyses were performed with IQ-TREE v. 2.2.0 (Nguyen et al. 2015) using the Zephyr v. 3.31 package (Maddison and Maddison 2023a) in Mesquite v. 3.81 (Maddison and Maddison 2023b). For both datasets, the concatenated UCE loci and the mitochondrial genomes, maximum-likelihood search was unpartitioned and used the TEST option (standard model selection followed by tree inference). For the maximum-likelihood tree, 10 search replicates were done; 1,000 bootstrap replicates were done.

Raw reads of new data are deposited in Sequence Read Archive (BioProject submission ID PRJNA1096354; Table 1). Alignments and trees are deposited in the Dryad data repository (https://doi.org/10.5061/dryad.hdr7sqvrf).

### ﻿Molecular phylogeny

3371 UCE loci were recovered initally, of which 199 were discarded for failing the branch-lengths paralogy test, and 1696 for being represented in too few taxa, leaving 1476 loci to be used in the analyses. In the trimmed, concatenated alignment the average sequence length is about 738,000 bp, though the *Hivanua* specimens are among the least well sequenced (average ~303,000 bp; Table 1).

For 10 of the taxa, between 12,700 and 14,480 bp of mitochondrial sequence (approximately the entire genome) was recovered as bycatch with the UCE-targetted reads (see column “mt bps” in Table 1). For three of the *Hivanua* specimens less was recovered (2568–4464 bp).

**Figures 1, 2.**
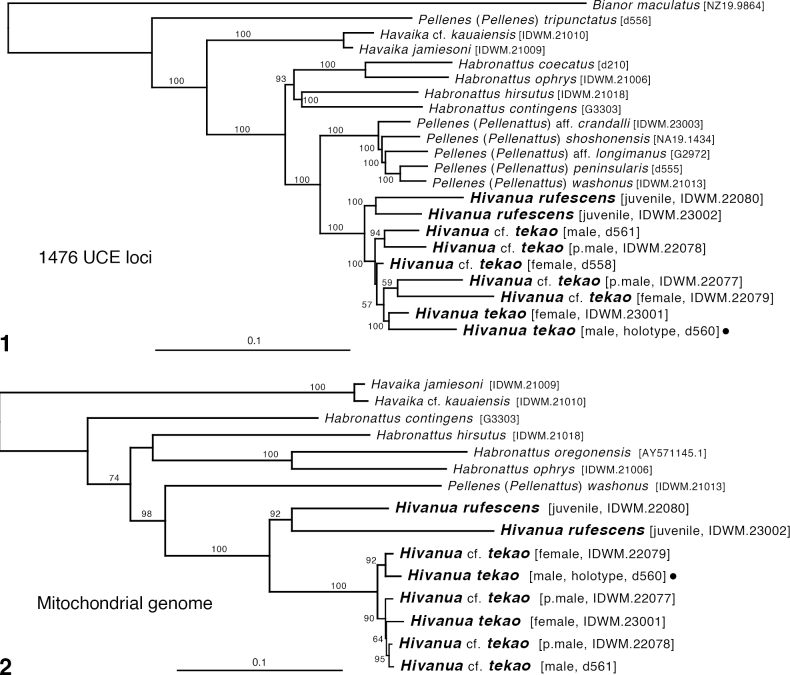
Phylogeny **1** maximum-likelihood tree from concatenated data set of 1476 UCE loci. **2** maximum-likelihood tree from mitochondrial genomes recovered as bycatch in UCE sequencing reads. Numbers are percentage of 1,000 bootstrap replicates showing the clade. Filled circle highlights the holotype of *Hivanuatekao* sp. nov.

In Figs 1, 2 are shown the maximum-likelihood phylogenies from 1476 UCE loci (Fig. 1) and from the mitochondrial genome (Fig. 2). *Hivanua* is strongly supported as sister group to the subgenus Pellenattus of *Pellenes*, with *Habronattus* their first cousin.

Within *Hivanua*, the nuclear UCE loci and the mitochondrial genome agree on the division between *Hivanua* specimens from Hiva Oa (*H.rufescens*, two specimens) and those from Nuku Hiva (*H.tekao* sp. nov. and H.cf.tekao, 6 specimens). However, interrelationships of specimens within Nuku Hiva are inconsistent, with (for example) male d560 strongly supported as sister to female IDWM.23001 by the concatenated UCE loci, but sister to female IDWM.22079 by the mitochondrial genome. The only agreed subclade is male d561 and subadult male IDWM.2078.

Of course, such conflict would be unsurprising if the six specimens of *H.tekao*/H.cf.tekao were conspecific, because one would expect there to be a networked pattern of genetic descent such that different parts of the genome would give different trees. However, the apparent morphological distinction of male d561 (discussed below) suggests there may be two species in the sample. If so, then the conflict among genomes could reflect incomplete lineage sorting or recent introgression. Although some clarity might be achieved by using coalescent methods of species delimitation (Knowles and Carstens 2007; Degnan and Rosenberg 2009; Yang and Rannala 2010; Smith and Carstens 2020), the paucity of specimens makes this unlikely to be informative. Thus, the conservative approach will be taken of naming, for now, only one species.

## ﻿Taxonomy

The molecular phylogeny’s strong placement of *Hivanua* species as sister group to PellenessubgenusPellenattus (Fig. 1) justifies their exclusion from the genera *Havaika* and *Habronattus*. While *H.tekao* sp. nov. and *H.rufescens* could be placed in an expanded *Pellenattus*, I establish for them a separate genus because of the drastically different embolus (long and thin), epigynal atria (of the ancestral crescent form), body form and setation, and habitat (lushly vegetated Pacific island).

The relatively sparse setation shared by *Hivanua* and *Havaika* may represent convergence towards a new microhabitat, living on foliage (see Natural History, below). Their ancestors, presumably open-ground dwellers like most other harmochirines, may have been especially suited to colonize new volcanic islands, but as the islands became vegetated, the spiders may have adapted to that new available microhabitat.

### ﻿ Hivanua

Taxon classificationAnimaliaAraneaeSalticidae

Maddison gen. nov.

DE40875F-DDC8-5160-9373-521C05D388AE

https://zoobank.org/26435A4C-485C-41E5-BA18-40592736CB81

#### Type species.

*Hivanuatekao* Maddison, sp. nov.

#### Species included.

*Hivanuaflavipes* (Berland, 1933), comb. nov., transferred from *Havaika*.

*Hivanuanigrescens* (Berland, 1933), comb. nov., transferred from *Plexippus*.

*Hivanuanigrolineata* (Berland, 1933), comb. nov., transferred from *Havaika*.

*Hivanuarufescens* (Berland, 1934), comb. nov., transferred from *Habronattus*.

*Hivanuatekao* Maddison, sp. nov.

*Hivanuatriangulifera* (Berland, 1933), comb. nov., transferred from *Havaika*.

#### Etymology.

An arbitrary combination of letters, containing a reference to the largest two islands of their range, Hiva Oa and Nuku Hiva. Grammatical gender: feminine.

#### Diagnosis.

Reflective scales and relatively sparse setation on the legs distinguish *Hivanua* and *Havaika* from other genera of the pellenine subgroup of harmochirines, which have fuller and more varied setation. *Hivanua* is distinct from *Havaika* by a more posterior placement of the epigynal coupling pocket (ECP). In *Hivanua*, the crescent-shaped atrial ridges shielding the openings reach posteriorly only as far as the midpoint of the ECP; in *Havaika*, the atrial ridges merge with the posterior end of the ECP (Prószyński 2002, 2008). Male first leg of *Hivanua* unusually long; for example, the holotype of *H.tekao* sp. nov. has a body length of 6.9 mm but a first leg length (femur to tarsus) of 12.5 mm. Third patella+tibia about the same length as fourth (distinctly longer in *Pellenes* and *Habronattus*). Palp with bulb smaller relative to cymbium and tibia compared to *Habronattus* and *Pellenes*. TmA sometimes present, unlike *Havaika*. First leg tibia usually or often with four anterior ventral macrosetae (other harmochirines with fewer). (Four macrosetae present in all *H.tekao*/H.cf.tekao, *H.flavipes*, *H.nigrolineata*, and about half of the *H.rufescens* specimens, mostly juveniles).

#### Species included.

**Figures 3–10.**
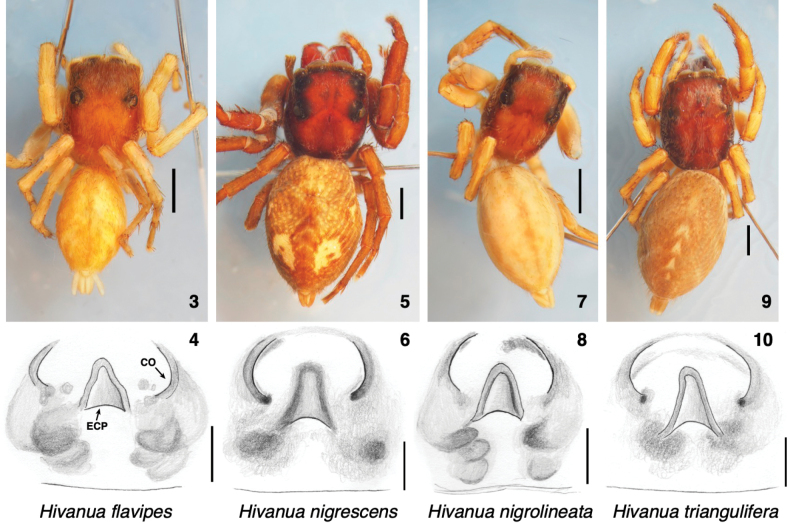
Berland’s holotypes of four *Hivanua* species, each showing habitatus and ventral view of epigyne **3, 4** holotype of *Sandalodesflavipes* Berland, 1933 **5, 6** holotype of *Sandalodesnigrescens* Berland, 1933 **7, 8** holotype of *Sandalodesnigrolineatus* Berland, 1933 **9, 10** holotype of *Sandalodestriangulifera* Berland, 1933. Abbreviations: CO, copulatory opening; ECP, epigynal coupling pocket. Scale bars: 1.0 mm for bodies; 0.1 mm for epigynes.

Six species are placed in *Hivanua*, five of which were described by Berland (1933, 1934). A new species, *H.tekao* sp. nov., is described below, and one of Berland’s, *H.rufescens*, is partially redescribed. The other four Berland species are not redescribed here except via illustrations of their female holotypes (in BPBM, examined; Figs 3–10). The holotype of *N.flavipes* is from “Hiva Oa, Mont Temetiu, 1300 m. d’alt.”, that of *H.nigrescens* is from “Tahuata: sommet du Haaoipu, 900 m”, that of *H.nigrolineata* from “Nukuhiva: Ooumu”, and that of *H.triangulifera* from Tahuata. There is variation among species in body form, with *H.nigrolineata* narrow and linearly marked, and *H.nigrescens* robust and with a rough texture. *H.nigrescens* was inexplicably synonymized with *Plexippuspaykulli* (Adouin, 1826) by Berland himself. The holotype of *H.nigrescens* is clearly a *Hivanua* (Figs 5, 6), similar to *H.tekao* sp. nov. One wonders if Berland confused it with his *Sandalodesmagnus* Berland, 1933, which is indeed a synonym of *P.paykulli*, and whose figures appeared in the same plate as *Sandalodesnigrescens*.

Several of Berland’s Marquesan harmochirines are placed in *Hivanua* only tentatively. *Hivanuarufescens* can be placed with the type species *H.tekao* sp. nov. with confidence based on the molecular evidence. These two species, along with *H.nigrescens* and *H.triangulifera*, are large bodied, distinctly larger than most of the Hawaiian *Havaika*. The remaining two species, *H.nigrolineata* and *H.flavipes*, are considerably smaller-bodied and more delicate, and could easily be mistaken for *Havaika*. They share with the larger *Hivanua* one distinction from *Havaika*, the more anterior placement of epigynal atria. For this, and for geographical parsimony, I will here place them into *Hivanua*, but this should be considered provisional until more material can be found and studied.

Species taxonomy of *Hivanua* is made difficult by the simplicity of the markings and genitalia, by the paucity of specimens, and by the fact that Berland’s type specimens are mostly female, harder to distinguish than males. Berland considered specimens from different islands as conspecific without good explanation. Adding to these difficulties is confusion over the geographic provenance of some specimens, mentioned under *H.rufescens* below.

### ﻿ Hivanua rufescens

Taxon classificationAnimaliaAraneaeSalticidae

(Berland, 1934) comb. nov.

5B49ADB3-4699-5F5B-B36C-CD5262D6DE75

Figs 11–14

Sandalodes rufescens Berland, 1934.
Habronattus rufescens —Prószyński 2002.

#### Diagnosis.

**Figures 11–14.**
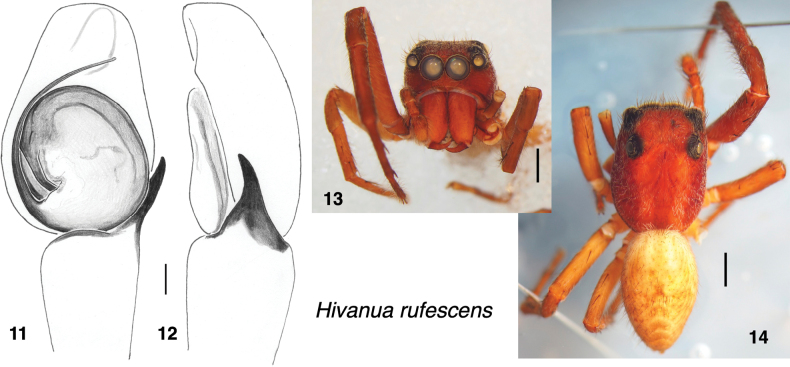
*Hivanuarufescens* (Berland, 1934), specimen IDWM.22076 from Hiva Oa. **11** palp, ventral view **12** same, prolateral **13** face **14** habitus. Scale bars: 0.1 mm for palp; 1.0 mm for body.

Similar to *H.tekao* sp. nov., large bodied, with long appendages, especially first legs in male, and light to medium brown throughout, except for indistinct markings. Distinguished from *H.tekao* sp. nov. by lack of TmA (Fig. 11).

#### Description.

**Male** (based on specimen IDWM.22076). Carapace length 3.95, width 2.86; abdomen length 3.8. ***Carapace*** (Fig. 14): slightly swollen at the cheeks, as if the cheliceral muscles are strong. Medium brown, with two longitudinal thoracic bands. ***Clypeus*** medium orange-brown, with a few white setae. ***Chelicerae*** vertical, orange-brown, with only sparse setae (Fig. 13). One simple retromarginal and two promarginal teeth. ***Palp***, like legs, uniformly brown with few setae. Patella and tibia unusually long compared to other pellenine harmochirines. Embolus thin, originating at about 7:30 (Fig. 11). Lacks TmA. ***Legs*** brown, front legs darker (medium rusty brown), back legs paler (light honey-brown). First legs especially long. First tibia with three anterior and three posterior ventral macrosetae. ***Abdomen*** indistinctly marked, with a trace of a central chevron.

**Female.** See Prószyński (2002).

#### Natural history.

The habitat is a “mountain ridge cloud forest” (Gillespie 2003). Although no specific notes were taken regarding the collecting methods for *H.rufescens*, the material listed below was bycatch of fieldwork seeking *Tetragnatha* and was most likely collected at night and from foliage (R. Gillespie pers. comm.).

#### Material examined.

1 male (IDWM.22076), 11 juveniles (including IDWM.22080, 23002) in EMEC with data French Polynesia: Marquesas Islands: Hiva Oa, Temetiu Ridge, 1170 m elev., 28-VI-2000, leg. R. Gillespie, G. Roderick. Gillespie (2003) reported this locality at 9.81°S, 139.08°W.

#### Remarks.

Prószyński (2002) did not provide any explanation for placing this species in *Habronattus*. A large and elbowed terminal apophysis (TmA) has been considered a synapomorphy of *Habronattus* (Maddison and Hedin 2003), but *H.rufescens* has no TmA (Arnedo and Gillespie 2006; Fig. 11). Nonetheless, Prószyński may have noticed some shared trait, because indeed *H.rufescens* is more closely related to *Habronattus* than to *Havaika*, and its congener *H.tekao* sp. nov. does have an elbowed TmA. The gap between the embolus and tegulum is larger in *H.rufescens* (Fig. 11) than in other Harmochirina lacking a TmA, as if leaving room for a TmA that was lost only recently.

There is some confusion about the geographic provenance of *H.rufescens* and perhaps also *H.tekao* sp. nov. Berland’s original description list the types of *H.rufescens* as from Nuku Hiva, but, as reported by Prószyński (2002), the labels with the type specimens in NHMUK (examined by D. Sherwood pers. comm.) indicate a collecting locality of Hiva Oa, 133 km to the southeast. Those specimens, studied by Prószyński (2002), do indeed appear to be the types, not only because his drawings match well Berland’s original drawings, but also because their labels seem clearly to be of the types. They read “Sandalodesrufescens Berland Type F et M”, and “1926.1.27.297-304; Hiva Oa, Marquesas Is.; C.L. Collenette 31.12.24; 3000–4000 ft.; S. Y. ‘St George’; S.E.R.A.” (D. Sherwood pers. comm.). This indicates the specimens were collected by the Scientific Expeditionary Research Association, from the ship “St George”, and formally accessioned by the NHMUK in 1926. While there could be an error in this label, it is more reasonable to respect the physical material and instead assume that Berland made an error in the publication.

The material collected more recently by Gillespie and others from Nuku Hiva and Hiva Oa (Arnedo and Gillespie 2006) could help resolve Berland’s confusion if it confirmed *H.rufescens* on Hiva Oa, except that there is unfortunately a similar contradiction between vial labels and published information for these more recent specimens. For a specimen they list as “*Habronattusrufescens*, Marquesas, Nuku Hiva”, Arnedo and Gillespie (2006) gave a palp photo (their figure 2Q) that can be matched to the specimen in EMEC here labelled IDWM.22076 (as indicated by the unusual dark line on the tegulum; Fig. 11). Its palp is a good match to the paratype of *H.rufescens* illustrated by Prószyński (2002). Thus, the specimen they reported as *H.rufescens* appears to be properly identified. However, that specimen and the accompanying juveniles are in vials whose labels indicate they were collected from Hiva Oa, not Nuku Hiva. Conversely, the other Marquesas male they discussed, “*Habronattus* sp. Marquesas, Hiva Oa” can be identified by details of setal placement (in their figure 2P) as specimen IDWM.22075 in a vial labelled as from Nuku Hiva. The DNA sequences they reported are likewise attributed to the correct species, but to the wrong islands. Those reported for *H.rufescens* (DQ531803 and DQ532084) are close matches to those obtained here from juveniles accompanying their *H.rufescens* male, while those sequences reported (DQ531801 and DQ532082) for the specimen that is here called *H.tekao* IDWM.22075 (their figure 2P) are closely similar to those obtained here from other specimens of *H.tekao* sp. nov. from Nuku Hiva. All of this is consistent with Arnedo and Gillespie attributing the palp and DNA to the correct specimens but recording their localities incorrectly. It is possible that Berland’s misreported locality for *H.rufescens* misled Arnedo and Gillespie to doubt and mistake the locality of their matching specimen.

I provisionally interpret the labeling of the vials to be correct for both the Berland and Gillespie specimens. The known specimens of *H.rufescens* are, therefore, from Hiva Oa. The male of *H.rufescens* that Arnedo and Gillespie showed in Fig. 2Q and whose DNA was reported as DQ531803, etc., is now labelled as specimen IDWM.22076. The known specimens of *H.tekao* sp. nov. are interpreted as from Nuku Hiva. The male shown in Arnedo and Gillespie’s figure 2P and whose DNA was reported as DQ531801, etc., is now labelled as specimen IDWM.22075.

### ﻿ Hivanua tekao

Taxon classificationAnimaliaAraneaeSalticidae

Maddison sp. nov.

55C3C7B8-C64C-5F36-9520-66266D6DF17E

https://zoobank.org//A213F09A-F764-411E-97D8-CD1447F742D8

Figs 15–22 , 27–30 ; possibly also Figs 23–26 , 31–38

#### Type material.

Male holotype (W. Maddison voucher code d560, in BPBM), with data French Polynesia: Marquesas Islands: Nuku Hiva, Mt Tekao, 1200 m elev. 23-VI-2000, leg. R. Gillespie. Female paratype (W. Maddison voucher code IDWM.23001, in EMEC), with data French Polynesia: Marquesas Islands: Nuku Hiva, Mt Tekao, 1185 m elev., 23-VI-2000, leg. R. Gillespie, L. Shapiro. Gillespie (2003) reported the 1185 m elevation locality at 8.86°S, 140.17°W. (See comments on provenance under *H.rufescens*.)

#### Etymology.

Derived from the name of the type locality; treated as a noun in apposition.

#### Diagnosis.

Embolus accompanied by a terminal apophysis (TmA), lacking in other species of *Havaika* and *Hivanua*. The TmA is long, thin, and elbowed, and thus resembles that of *Habronattus* (Griswold 1987). Otherwise, similar to *H.rufescens*, *H.tekao* sp. nov. is large bodied and with long appendages, especially the first legs in the male, and light to medium brown throughout except for indistinct markings. Females differ from those of *H.nigrolineata*, *H.flavipes*, and *H.triangulifera* in being more robust, with abdominal markings indistinct. Females are paler than the holotype of *H.nigrescens*. However, given the lack of clarity of which females belong to *H.tekao* sp. nov., any attempt to identify them is difficult at present.

#### Description.

**Figures 15–26.**
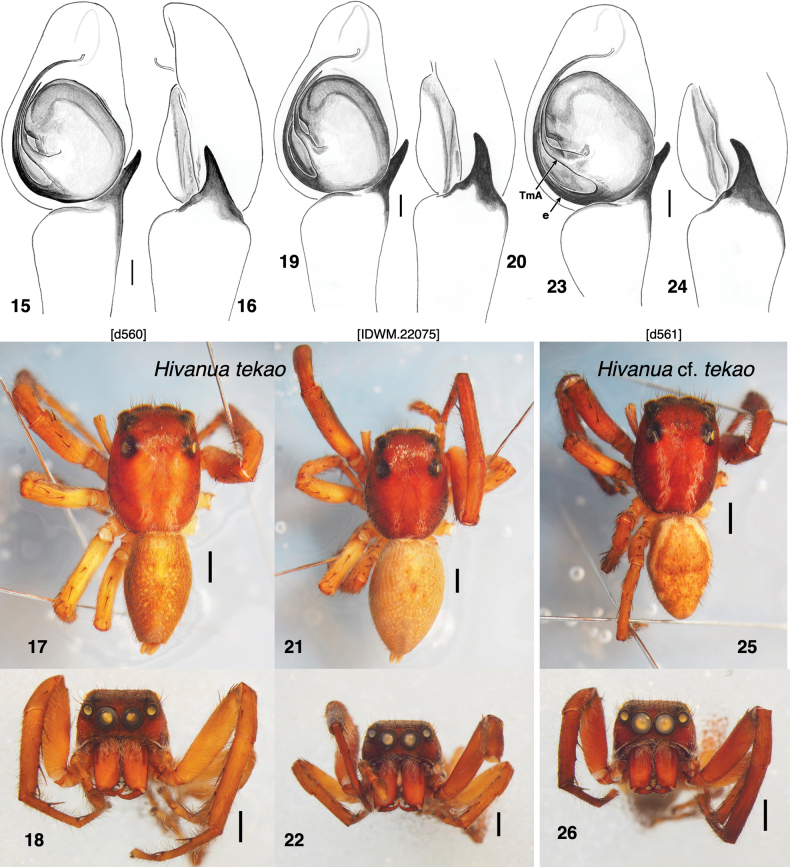
*Hivanuatekao* sp. nov. and a specimen that may be distinct, all from Nuku Hiva **15–18***H.tekao* sp. nov. holotype, specimen d560 **15** palp, ventral view **16** same, prolateral **17** habitus **18** face **19–22***H.tekao* sp. nov. male, specimen IDWM.22075 **19** palp, ventral view **20** same, prolateral **21** habitus **22** face. **23–26**H.cf.tekao, specimen d561 **23** palp, ventral view **24** same, prolateral **25** habitus **26** face. Abbreviations: TmA, terminal apophysis; e, embolus. Scale bars: 0.1 mm for palps; 1.0 mm for bodies.

**Male** (based on holotype, specimen d560). Carapace length 3.96, width 2.92; abdomen length 3.70. ***Carapace*** (Fig. 17): medium to dark brown, with two longitudinal thoracic bands of paler integument and thin covering of white scales, and marginal band of sparse white scales. Remainder of carapace thinly covered in setae, some in ocular quadrangle with bronze sheen. ***Clypeus*** medium to dark brown, sparsely covered with setae, with some long pale setae overhanging chelicerae (Fig. 18). ***Chelicerae*** vertical, orange-brown, with patch of white scales basally. One simple retromarginal and two promarginal teeth. ***Palp***, like legs, uniform brown with few setae. Patella and tibia unusually long compared to other pellenine harmochirines. Embolus thin, originating at about 7:00 (Fig. 15). TmA present, narrowing to a point, angled toward 10:30 initially, then bending (and thus elbowed) as it nears the embolus. ***Legs*** light brown, the front legs slightly darker. First legs especially long. First tibia with four anterior and three posterior ventral macrosetae. Length of femur I 3.65, II 2.29, III 2.76, IV 2.60; patella + tibia I 5.31, II 2.76, III 2.76, IV 2.71; metatarsus + tarsus I 3.54, II 2.14, III 2.66, IV 2.71. ***Abdomen*** indistinctly marked, with a trace of a central chevron.

**Figures 27–38.**
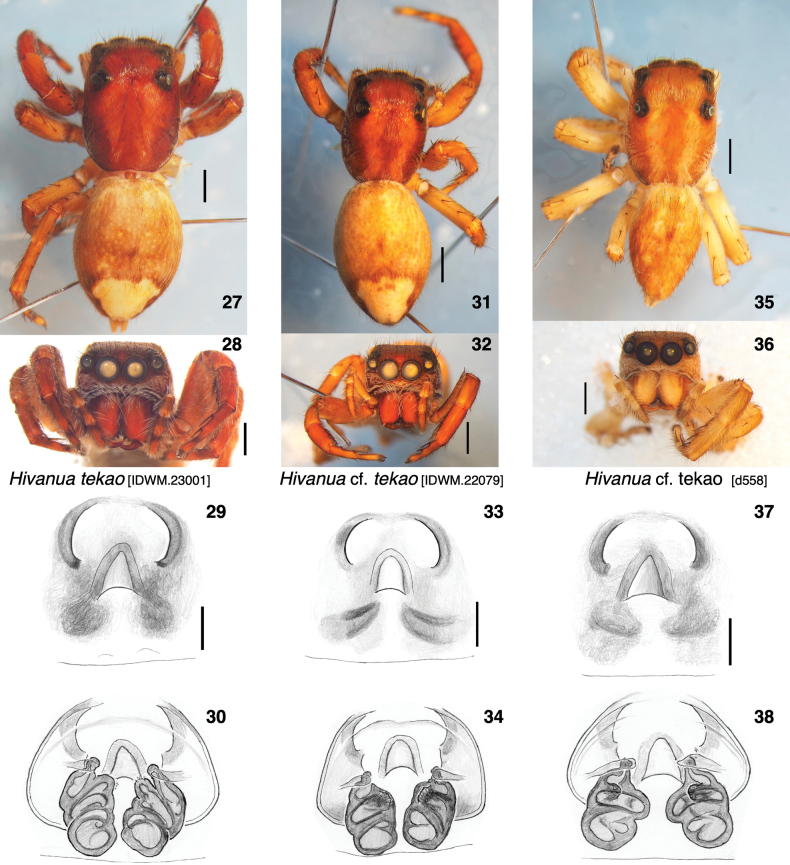
Females sequenced of *Hivanua* from Nuku Hiva, all *H.tekao* sp. nov. or a closely related species. Each shows habitatus, face, ventral view of epigyne, and dorsal view of cleared vulva **27–30** specimen IDWM.23001 **31–34** specimen IDWM.22079 **35–38** specimen d558. Scale bars: 1.0 mm for bodies; 0.1 mm for epigynes.

**Female** (based on specimen IDWM.23001). Carapace length 4.01, width 3.02; abdomen length 4.90. ***Carapace*** (Fig. 27): Structure, colour as in male. ***Clypeus*** brown covered with white scales, which overhang chelicerae. ***Chelicerae*** orange-brown, with a few white scales near the base. One simple retromarginal and two promarginal teeth. ***Legs*** light brown with a few scattered white scales. First tibia with four anterior and three posterior ventral macrosetae. Length of femur I 2.45, II 2.19, III2.60, IV 2.60; patella + tibia I 3.28, II 2.60, III 2.76, IV 2.76; metatarsus + tarsus I 2.08, II 1.77, III 2.40, IV 2.60. ***Abdomen*** medium brown except that posterior third is covered with a prominent triangular white patch, with white scales. ***Epigyne*** with basic simple *Habronattus* or *Bianor*-like form, with a triangular ECP placed centrally, flanked by two crescent-shaped atria (Fig. 29). The vulva shows the spermatheca forming a compact coil, much like those of *Habronattus* (Fig. 30).

#### Variation.

Three specimens can be reasonably securely considered to be *H.tekao* sp. nov. The male chosen as holotype, d560, closely resembles another male, IDWM.22075 in markings and palp; the female described, IDWM.23001, is placed next to the holotype in the phylogeny based on 1195 gene loci. The short branch lengths and discordance between mitochondrial and nuclear results (Fig. 1 vs. Fig. 2) would be consistent with those and all the other Mount Tekao specimens belong being a single species. However, on Mount Tekao there are two forms of males distinct enough that they might have been suspected as separate species. Male specimen d561 has longitudinal white stripes along the side of the abdomen and the embolus arising at 6:00, while males d560 and IDWM.22075 lack the stripes and have the embolus arising at about 7:00. Subadult male IDWM.22077 appears to match d561, with white stripes and 6:00 embolus origin (developed enough to see through the subadult integument), while subadult male IDWM.22078 lacks stripes and appears to have a 7:00 embolus origin (though this is unclear). The stripes could easily be polymorphic, but a difference in bulb rotation like that seen between Figs 15, 19 versus Fig. 24 would typically mark a different species, based on patterns in other groups. However, those differences do not form a clear pattern on the molecular phylogeny. From the UCE data (Fig. 1), one could suspect an unstriped less-rotated species (d560, IDWM.23001) and a striped more-rotated species (d561, IDWM.22077, 78, 79), but that would require doubting the appearance of the subadult IDWM.22078. It would also be contradicted by the mitochondrial genome, which places female IDWM.22079 instead of IDWM.23001 with the unstriped male d560. These two females appear morphologically the same, except that IDWM.23001 might have an extra coil in the spermatheca. That, however, would be against the expectations of IDWM.23001 belonging to a male with a shorter embolus. And, despite the difference in palp rotation, the striped male d561 has only one nucleotide difference with the unstriped male IDWM.22075 (Arnedo and Gillespie’s sequenced male from Nuku Hiva) in 16SND1.

Against all this confusion, I have decided to refer three specimens to *H.tekao* sp. nov. (male d560, male IDWM.22075, female IDWM.23001) and treat them as type material, and the remainder as possibly conspecific, naming them “H.cf.tekao”. Applying formal species delimitation methods to the UCE data might be able to help resolve it, but with so few specimens, it is prudent to wait until more specimens are available to determine if there is a second species.

#### Natural history.

The specimens from “above Toovii” are listed as “beated from ohia”. The others from Mount Tekao, including the type specimens, are not associated with specific habitat data. However, the specimens were likely on foliage. The type locality is a “high montane wet forest” (Gillespie 2003). The specimens were collected as bycatch of fieldwork seeking *Tetragnatha*, which was primarily at night and involved looking on foliage (R. Gillespie pers. comm.).

#### Additional material examined.

These are all identified only tentatively, as H.cf.tekao. The following are all in the EMEC, from French Polynesia: Marquesas Islands: Nuku Hiva. One male (voucher code d561), one subadult male (IDWM.22078) and 4 juveniles from Mt. Tekao, 1200 m elev., 23-VI-2000, leg. R. Gillespie. One male (IDWM.22075) and one subadult males (IDWM.22077) from Mt. Tekao, 1185 m elev., 23-VI-2000, leg. R. Gillespie, L. Shapiro. Two females (one is IDWM.22079) and one juvenile from Mt. Tekao, 1100 m elev., 24-VI-2000, leg. R. Gillespie. One female (d558) from Mt. Tekao, 1200 m elev., 25-VI-2000, leg. R. Gillespie. Two females and two juveniles from above Toovii, ~2800 ft., beaten from ohia, 18-vii-2001 Claridge.

#### Remarks.

The possibility that the specimens here described could be conspecific with one of Berland’s female holotypes should be addressed. The other holotype from Nuku Hiva, that of *H.nigrolineata*, is quite different, delicate bodied and with lineate markings. The male that Berland placed with *H.triangulifera* (not examined; location of specimen unknown) is from Nuku Hiva and could match that of H.cf.tekao shown in Figs 23–26, but there is no evidence to associate either of them with the female holotype of *H.triangulifera*, which is from another island, and differs from the females here considered to be *H.tekao* sp. nov. in having a simple clear chevron marking (the triangles of its specific epithet). The most obvious candidate for a match of *H.tekao* sp. nov. with a Berland species is with *H.nigrescens*, which, like *H.tekao* sp. nov., is large and robust. However, *H.nigrescens* is from a different and distant island, closer to Hiva Oa. The epigynes are too simple and poorly known to help. Because of the geographical distance, and to have a traceable name to which to attach the DNA data, the specimens here studied from Nuku Hiva are described as a new species.

## Supplementary Material

XML Treatment for Hivanua

XML Treatment for Hivanua rufescens

XML Treatment for Hivanua tekao

## ﻿Additional information

### Author ORCIDs

Wayne P. Maddison https://orcid.org/0000-0003-4953-4575

### Data availability

All of the data that support the findings of this study are available in the main text.
